# Effect of Kinesio Taping on the Walking Ability of Patients with Foot Drop after Stroke

**DOI:** 10.1155/2019/2459852

**Published:** 2019-05-15

**Authors:** Yilan Sheng, Shifeng Kan, Zixing Wen, Wenhua Chen, Qi Qi, Qiang Qu, Bo Yu

**Affiliations:** ^1^Department of Rehabilitation, Shanghai General Hospital, Shanghai Jiao Tong University, No. 100, Haining Road, Shanghai 200080, China; ^2^Department of Rehabilitation, School of International Medical Technology, Sanda University, No. 2727, Jinhai Road, Shanghai 201209, China; ^3^Department of Rehabilitation, Shanghai Fifth Rehabilitation Hospital, No. 279, Ledu Road, Shanghai 201600, China; ^4^Department of Rehabilitation, Shanghai Sunshine Rehabilitation Center, Yangzhi Affiliated Hospital of Tongji University, No. 2209, Guangxing Road, Shanghai 201209, China

## Abstract

**Objective:**

The purpose of this study was to investigate the effect of kinesio taping on the walking ability in patients with foot drop after stroke.

**Methods:**

Sixty patients were randomly divided into the experimental group (with kinesio taping) and the control group (without kinesio taping). The 10-Meter Walking Test (10MWT), Timed Up and Go Test (TUGT), stride length, stance phase, swing phase, and foot rotation of the involved side were measured with the German ZEBRIS gait running platform analysis system and were used to evaluate and compare the immediate effects of kinesio taping. All the measurements were made in duplicate for each patient.

**Results:**

The demographic variables of patients in both groups were comparable before the treatment (p>0.05). After kinesio taping treatment, significant improvement was found in the 10MWT and the TUGT for patients in the experimental group (p<0.05). There were significant differences in the 10MWT and TUGT between the experimental and control groups after treatment (p<0.05). In terms of gait, we found significant improvement in stride length (p<0.001), stance phase (p<0.001), swing phase (p<0.001), and foot rotation (p<0.001) of the involved side in experimental group after treatment compared with those before treatment. Further, the functional outcomes and gait ability were significantly improved in the experimental group after treatment (p<0.05), compared to the control group.

**Conclusion:**

Kinesio taping can immediately improve the walking function of patients with foot drop after stroke.

## 1. Introduction

Stroke, defined as the abrupt onset of a focal neurological deficit, is a leading cause of prolonged disability and death worldwide [1, 2]. Epidemiological investigations show that stroke survivors are at an increased risk of recurrent stroke, with 5- and 10-year estimates approximating 18% and 44%, respectively [3]. Most stroke survivors display a degree of motor dysfunction which affects the patient's daily activities, social participation, and quality of life [4]. Walking dysfunction is the most commonly reported limitation of the lower extremities in subjects after stroke [5], including the inability to dorsiflex the ankle, slow gait velocity, and increased risk for falls attributed to foot drop [6]. The current common treatment methods for rehabilitation include acupuncture [7], exercise therapy, physical therapy (such as functional electrical stimulation (FES)) [8], ankle-foot orthotics (AFO) [9], and kinesio taping [10].

Kinesio taping, also called elastic therapeutic tape or elastic sports taping [11], differs from other types of strapping tapes due to its unique elastic properties. In recent years kinesio taping technique is being popularly used in several health conditions. For instance, a study showed that the kinesio taping method may facilitate or inhibit muscle function and support joint structure for the upper extremity in hemiplegia in conjunction with other therapeutic interventions [12]. Kinesio taping application can also improve typical asymmetric gait and walking speed of the paralyzed parts of stroke patients [10]. Moreover, a recent study reported that the application of kinesio taping improved the center of pressure displacement and forward reach test results in stroke patients [13]. Therefore, in our study we suggest the use of kinesio taping for the treatment of foot drop after stroke.

With this purpose, this study examined the effect of kinesio taping on the walking ability in patients with foot drop after stroke. By assessing the immediate changes in walking before and after kinesio taping, the functional outcomes were compared with the control patients without kinesio taping. The use of kinesio taping for rehabilitation of foot drop after stroke might provide a theoretical basis and practical guidance.

## 2. Methods

### 2.1. Subjects

A total of 60 patients with foot drop after stroke recruited from Shanghai Sunshine Rehabilitation Center in China from September 2017 to June 2018 were enrolled in this study. All the patients provided informed consent before study participation. Patients who met the following criteria were included: (1) men and women aged 30–70 years; (2) patients who met the diagnostic criteria for stroke [14] and were diagnosed with stroke based on magnetic resonance imaging or computed tomography scan of the brain; (3) those who had a 3–12 month course of stroke; (4) those who were able to walk without assistance; and (5) those who had foot drop after stroke of the involved side (Modified Ashworth Scale (MAS) score [15]<2) without obvious contracture. The exclusion criteria were as follows: (1) patients who could not adhere to treatment regimens; (2) patients with stroke who had fluctuating progression; (3) patients with skin allergy and/or intolerance to tape, or acute musculoskeletal injury, or obvious skin lesions or swelling; (4) patients with serious and uncontrolled diseases such as coronary heart disease and uremia; and (5) patients with cognitive impairment on Mini-Mental State Examination (MMSE) score [16] < 27 points. Patients who were unable to complete experimental procedures due to allergies and other adverse events were also excluded.

The patients were randomized into two groups, experimental group (kinesio taping) and control group (without taping), based on random number generator in Stata 13.0 software [17]. A detailed characterization of these two groups is shown in Table 1.

### 2.2. Taping Intervention

**Figure 1:**
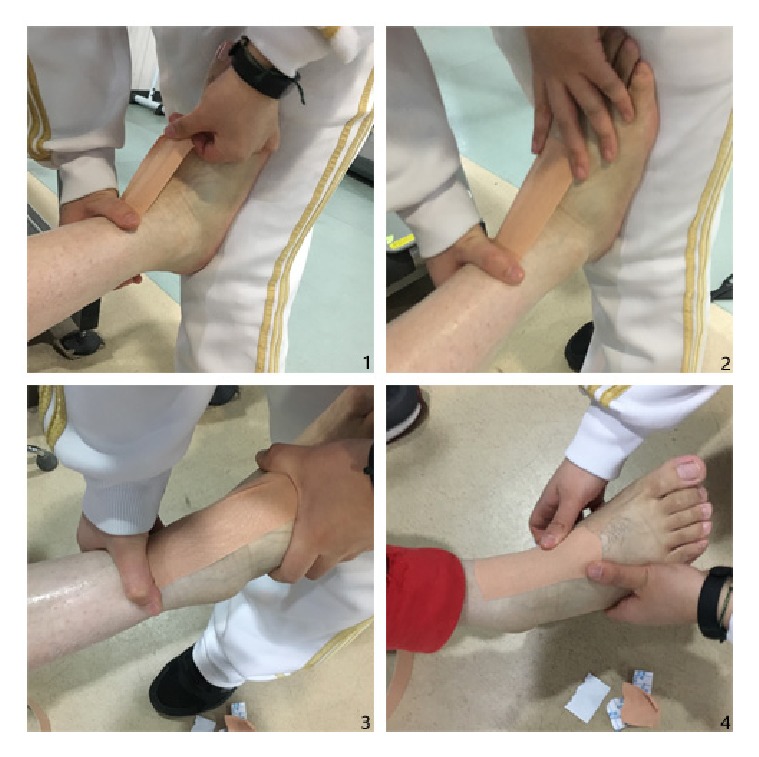
Kinesiology taping technique.

All kinesio taping treatments were performed by the same qualified physical therapist. Patients in both the groups underwent routine rehabilitation, including comprehensive training of the hemiplegic limb, exercise training on walking function, and different therapeutic modalities for foot drop. We used functional electrical stimulation (FES) for patients without ankle joint active range of motion (AROM) and electronic biofeedback therapy (EBFT) for patients with ankle joint AROM. For patients in the experimental group, the flesh-colored kinesio tape was used (Nanjing Syracuse Medical Products Co., Ltd.; product registration number: Suning Food and Drug Administration (quasi) Word 2011 No. 1640043; tape specification: size, 5 m × 5 cm (L × W)). Briefly, the tape was cut into an I-shaped patch of 5 cm × 20 cm, and both ends of the tape were attached onto the skin in the middle of the lower leg and the back of the foot, with dorsiflexion of the foot, while keeping the middle section of the taping suspended. The middle section of the tape was then attached firmly during the gradual flexion of the foot (Figure 1). The pulling force was 75% of the maximum tensile length of the tape [18]. Patients in the control group were not treated with taping.

### 2.3. Outcomes Assessment

**Figure 2:**
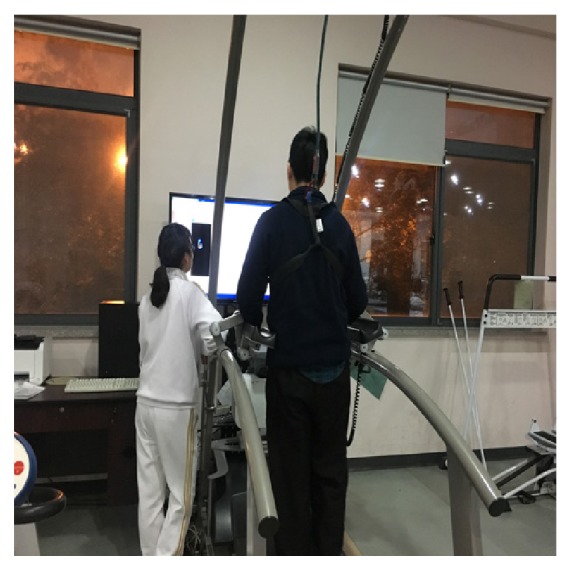
Assessments using the German ZEBRIS gait running platform analysis system.

The walking ability of the patients was evaluated before and after the taping. The evaluation parameters of the walking function included the 10-Meter Walking Test (10MWT), the Timed Up and Go Test (TUGT), stride length, stance phase, swing phase, and foot rotation of the involved side. The assessments were performed by a qualified physical therapist who was blinded to the experimental conditions. 10MWT was used to assess walking speed, whereby the time taken to walk 10 meters was recorded [19]. TUGT was used to assess the risk of falling and functional walking which recorded the time of standing from a back-rest chair, walking 3 meters, turning around a barrier, walking back to the back-rest chair, and sitting recumbently [20]. The stride length, stance phase, swing phase, and foot rotation of the involved side were evaluated by the German ZEBRIS gait running platform analysis system (zebris Medical GmbH, Max-Eyth-Weg 43, D-88316, Isny, Germany) (Figure 2) [21, 22]. To study the immediate effect of kinesio taping, the assessments were carried out immediately after taping. All the measurements were repeated twice for each patient.

### 2.4. Statistical Analysis

SPSS software (version 13.0) was used for the statistical analyses. Quantitative data were expressed as mean ± standard deviation. The paired sample t-test was used to compare the difference between the two groups before and after treatment. The comparison between the two groups was performed using the independent sample t-test. P<0.05 was considered statistically significant.

## 3. Results

### 3.1. Subject Characteristics

A summary of the general characteristics of the subjects is shown in Table 1. The experimental group contained 18 male and 12 female participants (mean age: 53.63±9.08 years; mean disease duration: 7.79±2.10 months). Of this, 15 cases had cerebral infarction and 15 cases had cerebral hemorrhage; 21 cases had left side involvement and 9 cases had right side involvement. Thirty subjects participated in the control group (20 males and 10 females; mean age: 51.89±8.48 years; mean course of disease: 7.91±2.65 months). Among them, 19 cases had cerebral infarction and 11 cases had cerebral hemorrhage; 14 cases had left side involvement and 16 cases had right side involvement. There were no significant differences in any of the demographic variables between these two groups (p>0.05). During the study period, 2 cases in the control group were excluded from the second assessment due to the patient's dissent from continuing in the study. No serious allergies or treatment-related complications occurred during the study.

### 3.2. Changes in Functional Outcomes and Gait Ability

After kinesio taping treatment, significant improvement was found in the 10MWT (41.17±2.41 to 37.28±2.89; p<0.001) (Table 2), and the TUGT (40.09±4.53 to 35.56±4.64; p<0.001) (Table 3) for patients in the experimental group. There were significant differences between the experimental and control groups after treatment with regard to the 10MWT and TUGT (Tables 2 and 3, p<0.05). In terms of gait, we found significant improvement in stride length (60.02±9.55 to 67.00±10.03; p<0.001), stance phase of the involved side (75.80±4.59 to 78.92±5.20; p<0.001), swing phase of the involved side (24.20±4.59 to 21.08±5.20; p<0.001), and foot rotation of the involved side (8.83±3.57 to 5.92±2.68; p<0.001) in experimental group after treatment compared with those before treatment (Table 4). Further, the functional outcomes and gait ability significantly improved in the experimental group after treatment (p<0.05) compared to the control group (Tables 2, 3, and 4).

## 4. Discussion

Foot drop after stroke is a common lower extremity motor dysfunction in stroke patients, which not only causes deformity of the foot and affects the appearance, but also affects the patient's standing, walking, and balance function to varying degrees [23]. After stroke, the weakness of the patient's tibialis anterior or spasm of the triceps of the calf muscle may cause oscillating and limited dorsiflexion, reduced loading of the lower limbs on the involved side, shortened support phase, and a shift in the center of gravity towards the healthy side. Further, the swing phase is prolonged, with a slower walking speed and reduced stride length. Clinical studies have pointed out that taping can improve extremity paralysis after stroke and assist in motor function rehabilitation training [10].

In our study, during the routine rehabilitation, the FES was applied for patients without ankle joint AROM, while the EBFT was adopted for patients with ankle joint AROM. The FES technique could use low energy electrical pulses to artificially generate foot movements in patients with foot drop after stroke. Based on reports, conventional physical therapy combined with FES reduced spasticity and improved the strength of ankle dorsiflexors and lower extremity motor recovery in stroke patients [24, 25]. EBFT had a long history of use in stroke rehabilitation. The study performed by Intiso et al. indicated that the EBFT increased muscle strength and improved the recovery of functional locomotion in patients with foot drop after cerebral ischemia [26]. Besides, drop foot after stroke may be addressed using Ankle-Foot Orthoses (AFO). Kluding et al. stated that there was significant improvement for patients using either an FDS or an AFO from baseline to 30 weeks in comfortable gait speed and fast gait speed, while there was no significant difference between FDS and AFO groups [9]. Based on the user experiences, more patients prefer to choose the FES rather than FDS [23]. Acupuncture has been reported to improve functional outcome after stroke. However, a study referring to acupuncture and transcutaneous nerve stimulation in stroke rehabilitation showed that treatment during the subacute phase of stroke with acupuncture had no beneficial effects on functional outcome or life satisfaction [7]. Kim et al. evaluated the changes in function and balance after kinesio taping application in stroke patients and the results showed that the kinesio taping had a positive effect on improvement of typical asymmetric gait and walking speed [10]. Our study evaluated the effects of kinesio taping on the rehabilitation of foot drop after stroke in a more systematic manner, and the results were consistent with the previous reports.

Under the premises of early intervention, the ankle joint dysfunction poststroke needs to be treated by proprioceptive training combined with biomechanics correction [27]. Reports have shown that taping can adjust the fascia, normalize muscle function, increase joint mobility, and improve joint stability [28]. A recent study indicated that temporary kinesiology taping positively improved static balance ability by increasing the Berg Balance Scale score and reducing the center of pressure in stroke patients with foot drop [29]. In this study, after application of kinesio taping for the patients in the experimental group, the 10MWT and TUGT immediately improved and decreased. Combined with the analysis of the gait, the stride length increased after taping, the proportion of the stance phase of the involved side increased, the swing phase accelerated, and the foot rotation of the involved side decreased. It can be concluded that after the kinesio taping, the walking ability and efficiency of the patient were immediately improved.

Kinesio taping can increase the range of motion and agility and improve the dorsiflexion function of the ankle by the elastic mechanism of the taping [30, 31]. The ankle joint sensation, which is closely related to balance and gait [32], is essential for the rehabilitation of walking function in stroke patients. Functional taping may stimulate the sensory input to the sensory receptor of the peri-ankle ligaments, improving the ankle joint flexion and extension, decreasing the foot rotation of the involved side, and stabilizing the overall posture control. Related studies have confirmed [13] that ankle joint taping can improve the posture control of stroke patients, thereby improving the functional activities such as balance and gait.

During the operation of functional taping, a large pulling force is applied to the taping which may be similar to the AFO that promotes the improvement of walking and balance function [33]. Previous studies have demonstrated that the use of AFO is conducive to the normal posture of stroke patients in the normal movement pattern during training through the accumulation of feedback for positive reinforcement to establish the correct body pattern [34]. In this study, kinesio taping could produce an immediate effect on correcting the position of the ankle, increasing the walking efficiency, and improving the walking function, which may assist with the development of related rehabilitation training.

However, this study had several shortcomings. For instance, there were differences in the walking function and related rehabilitation interventions in different patients. The additive effects of the subjects cannot be completely ruled out. The data measured by the German ZEBRIS gait running platform analysis system is limited, and there is still a lack of high-quality literature to support the accuracy of its data capture and analysis. Therefore, in this experiment, the physical therapist simply referred to the relevant clinical research and selected some gait spatial and temporal parameters to be compared before and after the intervention.

## 5. Conclusion

In conclusion, our study suggested that for patients with foot drop after stroke, the use of kinesio taping may help to improve posture control as well as exercise patterns and instantly produce immediate effects on walking and balance. Randomized control studies and analysis of influencing factors in future can provide more theoretical basis and practical support for the application of kinesio taping effects in the field of neurological rehabilitation.

## Figures and Tables

**Table 1:** General characteristics of subjects.

Parameters	EG (n=30)	CG (n=30)	Χ^2^/t	P-value
Gender	18/12 (60.00%/40.00%)	20/10 (66.67%/33.33%)	0.287	0.592
Male/Female (%)				
Age, years	53.63 ± 9.08	51.89 ± 8.48	0.765	0.447
BMI, kg/*m*^2^	24.23 ± 3.08	23.97 ± 2.50	0.353	0.725
Course of Disease, Months	7.79 ± 2.10	7.91 ± 2.65	0.200	0.842
Involved side	21/9 (70.00%/30.00%)	14/16 (46.67%/53.33%)	3.360	0.067
Left/Right(%)				
Types of Stroke	15/15 (50.00%/50.00%)	11/19 (36.67%/63.33%)	1.086	0.297
Cerebral hemorrhage/Cerebral infraction(%)				

Data are expressed as n (%) or mean ± standard deviation. EG, experimental group; CG, control group.

**Table 2:** Comparison of 10MWT within groups and between groups.

Parameter		EG (n=30)	CG (n=28)	t	P-value
10MWT(sec)	Pre	41.17 ± 2.41	40.85 ± 3.43	0.415	0.680
	Post	37.28 ± 2.89	40.03 ± 3.43	3.315	0.002^*∗*^
	t	11.709	6.474		
	*P-value*	<0.001^*∗*^	<0.001^*∗*^		

Data are expressed as mean ± standard deviation. ^*∗*^ p<0.05. EG, experimental group; CG, control group; Pre, before experiment; Post, after experiment.

**Table 3:** Comparison of TUGT within groups and between groups.

Parameter		EG (n=30)	CG (n=28)	t	P-value
TUGT(sec)	Pre	40.09 ± 4.53	40.32 ± 4.52	0.194	0.847
	Post	35.56 ± 4.64	39.88 ± 4.52	3.592	0.001^*∗*^
	*t*	8.434	3.335		
	*P-value*	<0.001^*∗*^	0.002^*∗*^		

Data are expressed as mean ± standard deviation. ^*∗*^ p<0.05. EG, experimental group; CG, control group; Pre, before experiment; Post, after experiment.

**Table 4:** Comparison of the stride length, as well as stance phase, swing phase of the involved side, and foot rotation of the involved side within groups and between groups.

Parameter		EG (n=30)	CG (n=28)	t	P-value
SL (cm)	Pre	60.02 ± 9.55	57.70 ± 9.40	0.932	0.356
	Post	67.00 ± 10.03	58.10 ± 9.39	3.484	0.001^*∗*^
	*t*	26.277	4.621		
	P-value	<0.001^*∗*^	<0.001^*∗*^		
STP(%)	Pre	75.80 ± 4.59	74.69 ± 6.39	0.766	0.447
	Post	78.92 ± 5.20	75.55 ± 6.28	2.234	0.029^*∗*^
	*t*	8.506	6.982		
	P-value	<0.001^*∗*^	<0.001^*∗*^		
SWP (%)	Pre	24.20 ± 4.59	25.31 ± 6.39	0.766	0.447
	Post	21.08 ± 5.20	24.45 ± 6.28	2.234	0.029^*∗*^
	*t*	8.506	6.982		
	P-value	<0.001^*∗*^	<0.001^*∗*^		
FR	Pre	8.83 ± 3.57	9.58 ± 2.94	0.870	0.388
	Post	5.92 ± 2.68	8.94 ± 2.85	4.155	<0.001^*∗*^
	*t*	9.028	6.174		
	P-value	<0.001^*∗*^	<0.001^*∗*^		

Data are expressed as mean ± standard deviation. ^*∗*^ p<0.05. Abbreviation: EG, experimental group; CG, control group; Pre, before experiment; Post, after experiment; SL, stride length; STP, stance phase of the involved side; SWP, swing phase of the involved side; FR, foot rotation of the involved side.

## Data Availability

Individual participant data will be available. All of the individual participant data were collected during the trial, after assessment, by certificated physical therapist. All the data are available for anyone who wishes to access them for any analysis purpose immediately following publication and with no end date. The parameters related to the general characteristics of subjects and the results of assessments before and after the trial used to support the findings of this study are included within the article. The evaluation parameters of the walking function included the 10-Meter Walking Test (10MWT), the Timed Up and Go Test (TUGT), stride length, stance phase, swing phase, and foot rotation of the involved side.
